# DDX42 Enhances Hepatocellular Carcinoma Cell Proliferation, Radiation and Sorafenib Resistance via Regulating GRB2 RNA Maturation and Activating PI3K/AKT Pathway

**DOI:** 10.1111/jcmm.70793

**Published:** 2025-08-19

**Authors:** Zijian Liu, Jingsheng Yuan, Fei Liu, Qiwen Zeng, Zhenru Wu, Jian Yang

**Affiliations:** ^1^ Laboratory of Liquid Biopsy and Single Cell Research, Cancer Center, West China Hospital Sichuan University Chengdu Sichuan China; ^2^ Department of Radiation Oncology, Cancer Center, West China Hospital Sichuan University Chengdu Sichuan China; ^3^ Department of Head and Neck Oncology, Cancer Center, West China Hospital Sichuan University Chengdu Sichuan China; ^4^ Liver Transplant Center, Transplant Center, West China Hospital Sichuan University Chengdu Sichuan China; ^5^ Laboratory of Liver Transplantation, Frontiers Science Center for Disease‐Related Molecular Network West China Hospital of Sichuan University Chengdu Sichuan China; ^6^ Division of Biliary Tract Surgery, Department of General Surgery, West China Hospital Sichuan University Chengdu Sichuan China; ^7^ Laboratory of Pathology, Key Laboratory of Transplant Engineering and Immunology, NHC West China Hospital of Sichuan University Chengdu Sichuan China

**Keywords:** cell proliferation, hepatocellular carcinoma, radio‐resistance, RNA maturation, sorafenib resistance

## Abstract

The DEAD‐box RNA helicase (DDX) family is one of the canonical splicing regulators, engaged in RNA metabolism, and generally participates in forming spliceosomes. However, systematic analysis of DDX family members in hepatocellular carcinoma (HCC) has not been conducted before, and their biological functions need to be investigated further. Based on biological function enrichment analysis, radiosensitivity index (RSI), and prediction IC50 index for sorafenib, we ultimately ascertain DDX42 as a candidate gene. DDX42 was highly expressed in HCC than in para‐tumour tissues and was a prognostic factor for HCC patients. Importantly, DDX42 overexpression promotes cell proliferation, radio‐resistance and sorafenib resistance in HCC cells and activates the PI3K/AKT pathway. Knockdown of DDX42 moderately inhibited cell growth of HCC cells and significantly increased radio‐sensitivity, enhanced the efficacy of sorafenib, and inactivated the PI3K/AKT pathway. Mechanically, DDX42 could urge the mRNA maturation of GRB2, contributing to cell proliferation and enhancement of resistance ability to radiotherapy and sorafenib for HCC cells. Subcutaneous xenograft nude mouse model showed that DDX42 significantly promoted tumour growth as compared to the control group and lifted the expression of GRB2, KI‐67 and PCNA in vivo. In conclusion, our findings facilitate the acknowledgment of tumour initiation and mechanisms of treatment resistance in HCC, and targeting the axis of DDX42 and GRB2 may be promising strategies for synergy with radiotherapy or sorafenib for HCC patients.

**Abbreviations**
CCK‐8: Cell counting kit‐8
DAVID: Database for Annotation, Visualisation and Integrated Discovery
DDX: DEAD‐box RNA helicase
DFS: Disease‐free survival
EdU: 5‐Ethynyl‐2′‐deoxyuridine
GEO: gene expression omnibus
GO: gene ontology
GRB2: growth factor receptor bound protein 2
GSVA: gene set variation analysis
GTEx: genotype‐Tissue Expression
HCC: hepatocellular carcinoma
HR: hazard ratios
IC50: half maximal inhibitory concentration
ICGC: International Cancer Genome Consortium
IHC: immunohistochemistry
KEGG: Kyoto Encyclopedia of Genes and Genomes
mRNAsi: gene expression‐based stemness index
NMF: non‐negative matrix factorisation
OS: overall survival
PDC: proteomic data commons
PFS: progression‐free survival
qRT‐PCR: real‐time quantitative PCR
RSI: radiosensitivity index
TCGA: The Cancer Genome Atlas
*t*‐SNE: *t*‐distributed stochastic neighbour embedding
WB: western blot

## 1 Introduction

Hepatocellular carcinoma (HCC) is the most common pathological type of primary liver malignancies, ranking sixth in morbidity and third in mortality in the world [1]. Due to the insidious onset of HCC and the lack of specific early clinical symptoms, most patients are already in the advanced stages when diagnosed, resulting in a low success rate of radical operation [2]. Local and systemic treatment strategies have improved the prognosis of advanced HCC patients, but a large proportion of them still suffer treatment failure due to tumour progression and treatment resistance [3]. Radiotherapy (RT) [4] and targeted therapy [5] are the few therapies with proven clinical feasibility for HCC patients, but their effectiveness has been constrained due to primary or acquired resistance. Therefore, it is very urgent to explore the potential targets or molecular mechanisms of tumour progression and treatment resistance for HCC, which might help to improve the efficacy of HCC treatment and the overall survival rate of HCC patients.

Benefiting from data re‐analysing with public datasets, a better understanding of members involved in diverse gene families for specific tumour types in stakes of biological function is gradually obtained. Dysregulation of mRNA maturation processes in multiple types of malignancies has been proven to be correlated with tumour progression, treatment resistance, and unfavourable prognosis [6]. The DEAD‐box RNA helicase (DDX) family is one of the canonical splicing regulators, engaged in RNA metabolism, and generally participates in forming spliceosomes [7]. Several studies have shown that abnormal expression of DDX family members is associated with carcinogenesis, such as DDX17, which promotes HCC metastasis by regulating the alternative splicing of PXN‐AS1 [8]. It has been reported that DDX56 could transcriptionally activate MIST1 to facilitate tumorigenesis of HCC through the PTEN/AKT signalling pathway [9]; DDX1 could inhibit CD8+ T cell antitumour activity by inducing PD‐L1 expression in HCC [10]. However, systematic analysis of DDX family members in HCC has not been conducted before; there are still diverse biological functions involved in tumour progression and treatment resistance, including radio‐ and sorafenib resistance, which need to be investigated.

Resorting integrated analyses from multi‐omics with public datasets, we systematically elucidated the underlying biological function and clinical characteristics of DDX family members in HCC, and DDX42, which had never been studied before in HCC, could play antiviral effects in various cell types [11] and take part in spliceosome assembly processes with DDX46 [12]. In this research, DDX42 was found to be highly associated with tumour progression, acquired radio‐resistance and sorafenib resistance in HCC. Mechanically, we found GRB2, which was an adaptor protein in the upstream of receptor tyrosine kinase signalling cascade [13], providing a key function in PI3K/AKT signal transduction [14], could be regulated by DDX42 by affecting the processes of RNA maturation. Prior studies showed that aberrant expression of GRB2 could influence the invasion and metastasis abilities of HCC cells [15]. At present, there were no reports about the association between GRB2 and DDX42 regarding regulating tumour progression and treatment resistance.

In this study, we first conducted clustering analyses about DDX family members in transcriptome levels to divide them into two separate groups, called DDX groups, exhibiting significantly different survival conditions and preponderant biological pathways. Based on the *t*‐distributed stochastic neighbour embedding (*t*SNE) method, the *t*SNE score was calculated with the expression of DDX family members to quantify the biological characteristics of the two DDX groups. We leveraged the gene expression‐based stemness index (mRNAsi), radiosensitivity index (RSI), prediction half maximal inhibitory concentration (IC50) of sorafenib, and ESTIMATE score to conduct biological‐related assessments. Additionally, we demonstrated that the expression differences between tumour and normal tissues of DDX42 and GRB2 in HCC specimens via IHC experiments. We further validated the role of DDX42 and GRB2 in cell proliferation, and radio‐ and sorafenib resistance in HCC cells via clonogenic, CCK‐8, and EdU assays, and identified their potential downstream PI3K/AKT signalling pathways. Furthermore, we reported that overexpression of DDX42 could alter the mRNA maturation of GRB2. Moreover, we validated the function of tumour progression enhancement for DDX42 in vivo experiments, underscoring its potential as a diagnostic and therapeutic target for HCC.

## 2 Methods and Materials

### 2.1 Public Data Processing

The transcriptome and clinical raw data of HCC from The Cancer Genome Atlas (TCGA) and International Cancer Genome Consortium (ICGC) datasets were downloaded from the UCSC XENA database (https://xena.ucsc.edu/). Proteomics data and clinical traits for HCC were obtained from supplementary data in a previous study [16]. Additionally, the expression matrix for dataset GSE207288 was directly downloaded from the GEO database. Basic expression levels of DDX family members and GRB2 between HCC and normal liver tissues from TCGA and Genotype‐Tissue Expression (GTEx) datasets were downloaded from Sanger Box (https://www.sangerbox.com). Furthermore, IHC representative images of DDX42 and GRB2 between HCC and normal tissues were directly downloaded from the Human Protein Atlas (HPA) database (https://www.proteinatlas.org).

### 2.2 Identification of Molecular Subtypes and Construction of *t*SNE Score

To investigate the molecular subtypes of HCC, non‐negative matrix factorisation (NMF) analysis was conducted to cluster samples with the expression of DDX family members. The cophenetic, dispersion and silhouette indicators were used to determine the optimal clustering number; the optimal number of clusters was set at 2 based on the most significant drop in cophenetic values. We utilised the *t*‐distributed stochastic neighbour embedding (*t*SNE) analysis to construct a *t*SNE score, similar to the calculation method of the PCA score in our previous study [17], and the *t*SNE score was calculated as follows: *t*SNE score = Σ (*t*SNE1_*i*_ + *t*SNE2_*i*_).

### 2.3 Biological Functional Enrichment Analysis

Kyoto Encyclopedia of Genes and Genomes (KEGG) and gene ontology (GO) analyses were conducted in The Database for Annotation, Visualisation and Integrated Discovery (DAVID) database (https://david.ncifcrf.gov/tools.jsp). Visualisation of biological function and intersection results of feature genes among hepatocyte clusters was performed using the imageGP database (http://www.bic.ac.cn/ImageGP). To quantitatively assess biological function, gene set variation analysis (GSVA), a non‐parametric and unsupervised method, was conducted using the ‘GSVA’ package in R with two validated gene signatures [18, 19]. Gene expression and gene signature enrichment scores were visualised in a heatmap plot in R using the ‘pheatmap’ package.

### 2.4 Cell Culture

Human HCC cell lines (HepG2, Huh7, PLC5, LM3, Hep1 and Hep3B) and normal adult liver epithelial cells (THLE2 and THLE3) were purchased from the Cell Bank of the Chinese Academy of Sciences (Shanghai, China). All cell lines were cultured in DMEM (Gibco, NY, USA) supplemented with 10% fetal bovine serum (FBS) (ScienCell, CA, USA) and 1% penicillin and streptomycin (HyClone, UT, USA) and grown at 37°C in humidified air with 5% CO_2_. All cell lines were examined by short tandem repeat profiling for cell line authentication and routine mycoplasma detection.

### 2.5 Animal Experiments

Six‐week‐old female BALB/c nude mice were purchased and fed as described before [20]. All operations on laboratory animals were performed following the NIH Guide for the Care and Use of Laboratory Animals and were approved by the Animal Care and Use Committee of West China Hospital, Sichuan University (2020351A). LM3 cells with designed treatment were collected and injected subcutaneously into mice in different groups with at least five mice in each group. Tumour growth was monitored every other day, and the mice were euthanised 2 weeks after injection. Each tumour was dissected, weighed, fixed with 4% formaldehyde, and embedded in paraffin. The formula for calculating tumour volume was as follows: volume = 1/2 × longest diameter × (shortest diameter)^2^.

### 2.6 Clinical Samples and Immunohistochemistry (IHC)

Ten paired fresh human HCC tissues with para‐tumour tissues from West China Hospital of Sichuan University were used in this study. IHC staining was conducted on clinical samples and mouse xenograft tumours as described previously [21]; the IHC results were evaluated by two independent observers.

### 2.7 Lentivirus Construction and Transfection

HEK293T cells were co‐transfected with packaging vectors psPAX2 and pMD2G and the DDX42 lentiviral plasmid using a standard protocol. They were selected with puromycin (Biofroxx, #1299MG025) for 2 weeks to generate stable cell lines [22]. The vectors of pLV3‐puro were all purchased from MiaoLingBio, China. The full‐length open reading frames of DDX42 were amplified respectively by RT‐PCR and cloned into the vectors. The small interfering RNAs (siRNAs) targeting the coding region were constructed based on the following sequences:

siDDX42‐1: 5′‐CUUACCUUGUGUUUGAUGA‐3′;

siDDX42‐2: 5′‐CAGAAUGCCUGGUUUCGGA‐3′;

siGRB2: 5′‐CAUGUUUCCCCGCAAUUAUTT‐3′.

### 2.8 Colony Formation

For clonogenic cell survival assays, cells were centrifuged and resuspended in a medium, and 1000 cells were seeded in a 12‐well petri dish. After 10 days of seeding, colonies were fixed with paraformaldehyde (4%) and stained with 0.1% crystal violet. Colonies were defined as clusters of > 50 cells and counted using Image J.

### 2.9 Radiation Clonogenic Assay

Cells were added to a six‐well culture plate at a density of 400, 400, 800, 2000, 4000 cells per well and exposed to IR at dosages of 0, 2, 4, 6, and 8 Gy, respectively. Colony numbers were counted as described above, and the surviving fraction (SF) curve was revised via a multi‐target single‐hit model with the following formula: SF = 1 − (1 − *e* ^−D/D0^)^*N*^.

### 2.10 Cell Counting Kit‐8 (CCK‐8)

The proliferation of cells in the indicated group was measured using the CCK‐8 assay according to the manufacturer's instructions. Five thousand HCC cells were seeded in a 96‐well plate, and then cells were harvested and detected using the CCK‐8 kit. Briefly, 10 μL CCK‐8 solution and 90 μL DMEM medium were premixed and added to each well, and absorbance at 450 nm was measured after 2 h incubation. Each cell group was plated in five duplicate wells.

### 2.11 5‐Ethynyl‐2′‐Deoxyuridine (EdU) Assays

To explore the effect of UBAP2 and SLC27A5 on the proliferation capacity of HCC cells, HCC cells were seeded in a 96‐well plate with a cell density of 1 × 10^4^/mL. The cells were cultured for 24 h, stained with Alexa Fluor 594 (Beyotime, Wuhan, China) according to the manufacturer's instructions, and observed using a fluorescence microscope (Olympus, Japan).

### 2.12 Real‐Time Quantitative PCR (qRT‐PCR)

Total RNA was extracted using TRIzol reagent (Invitrogen, USA), and cDNA was synthesised using a High‐Capacity cDNA Reverse Transcription Kit (Takara, Japan) according to the manufacturer's instructions. The mRNA level of DDX42 and GRB2 was measured using the TBGreen kit (Takara, Japan), and the 2^−ΔΔCT^ method was used for fold change calculation, normalised to GAPDH. Primer sequences were listed:

DDX42 forward: 5′‐GGCCTATACCCTACTCACTCCC‐3′;

DDX42 reverse: 5′‐CCACCAATGTTCAGCTTTTTTCC‐3′;

GRB2 forward‐1: 5′‐ACAGACCAGGAATGCAATGT‐3′;

GRB2 reverse‐1: 5′‐CCAGCTCTCCATCCTCCT‐3′;

GRB2 forward‐2: 5′‐GTCTCCAGAAACCAGCAGATATT‐3′;

GRB2 reverse‐2: 5′‐CCAGCTCTCCATCCTCCT‐3′;

GAPDH forward: 5′‐ACCCAGAAGACTGTGGATGG‐3′;

GAPDH reverse: 5′‐CAGTGAGCTTCCCGTTCAG‐3′.

### 2.13 Western Blotting

Total proteins extracted from HCC cells were subjected to sodium dodecyl sulphate‐polyacrylamide gel electrophoresis, and western blotting was conducted as described before [23]. An anti‐GAPDH antibody was used for the normalisation of protein expression. Signals were detected using enhanced chemiluminescence (Meilunbio, Dalian, China). The main primary antibodies used are anti‐DDX42, anti‐GAPDH (Hangzhou HuaAn biotechnology, China) and anti‐GRB2, anti‐Ki67, anti‐PCNA (Proteintech, China), anti‐AKT, anti‐PI3K, anti‐phospho‐AKT and anti‐phospho‐PI3K (Cell Signalling Technology, America).

### 2.14 Statistical Analysis

Correlation coefficients and *p*‐values were determined by Spearman correlation analysis. The Kaplan–Meier method was used to calculate the survival rate, with the log‐rank test for significance. The univariate Cox regression model was employed to calculate the hazard ratios (HR) for candidate input genes. The normal distribution of the data was assessed by the Kolmogorov–Smirnov test. For normally distributed data, the student's *t*‐test was used for two‐group comparisons, and one‐way ANOVA for multiple‐group comparisons. For non‐normally distributed data, Kruskal–Wallis tests were used to compare differences among more than two groups, and Wilcoxon tests to compare differences between two groups. The chi‐square test was used to analyse the clinical correlation between gene expression and clinicopathological features. All statistical *p*‐values were two‐sided, with *p* < 0.05 considered statistically significant.

## 3 Results

### 3.1 Consolidated Analysis for DDX Family Genes in HCC Based on Bioinformatics

**FIGURE 1:**
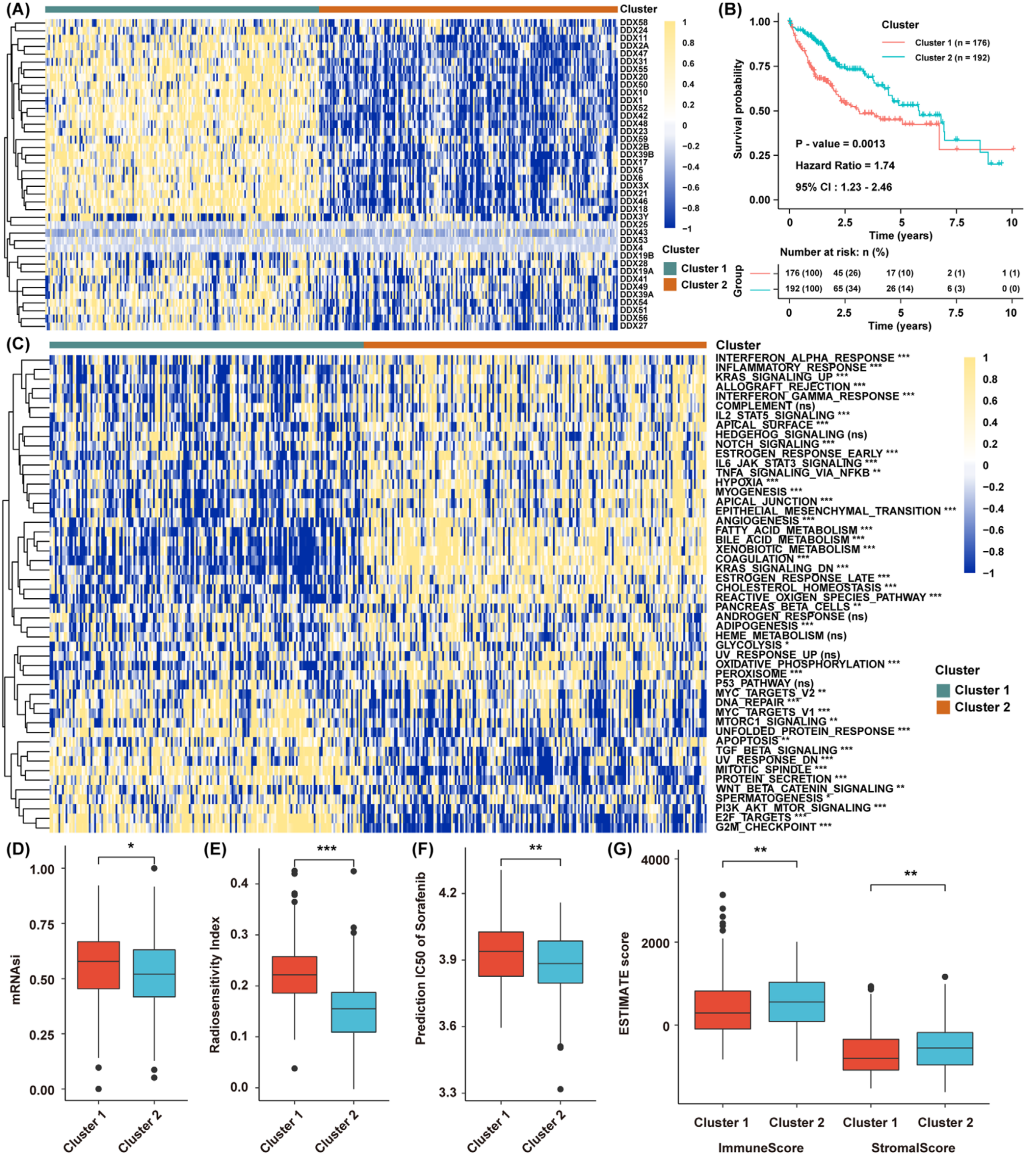
Consolidated analysis for DDX family genes in HCC based on bioinformatics. (A) The mRNA expression levels of DDX family members of two clusters in TCGA. (B) Survival analysis of two clusters in HCC. (C) Pathway enrichment scores between the two clusters. (D–G) The comparison of mRNAsi, RSI, prediction IC50 of sorafenib and ESTIMATE score between the two clusters. The asterisks represent the statistical *p*‐value (*p < 0.05, **p < 0.01, ***p < 0.001, ns, no significance).

Based on the transcriptional expression of DDX family members in the TCGA HCC cohort, we resorted to the NMF clustering method to divide HCC samples into distinct groups to explore the underlying biological function of DDX family genes (Figure S1). As the cophenetic within the NMF rank survey began to decline, we set *k* = 2 as the condition and separated the DDX family members into two significantly varied groups, indicating that this grouping method might reflect the deeper correlation among these genes (Figure 1A). Survival analysis showed that the prognosis of cluster 1 was significantly worse than that of cluster 2 (Figure 1B), suggesting that higher expression of DDX family members might correlate with a worse prognosis. GSVA showed that significant pathway differences existed between the two clusters (Figure 1C), and several cell survival‐related pathways, such as MYC, E2F, TGF‐β, G2M checkpoint and PI3K/AKT pathways, were highly enriched in cluster 1, while immune‐related pathways, interferon‐α/β response, IL2/STAT5, and IL6/JAK/STAT3 were highly enriched in cluster 2. This meant that cluster 1 might be associated with aggressive tumour progression and treatment resistance phenotypes, while cluster 2 might be correlated with a better immune response. To verify the posit, we found that the mRNAsi, RSI, and prediction IC50 of sorafenib were higher in cluster 1 than in cluster 2 (Figure 1D–F), while the immune and stromal scores were higher in cluster 2 than in cluster 1 through ESTIMATE analysis (Figure 1G).

**FIGURE 2:**
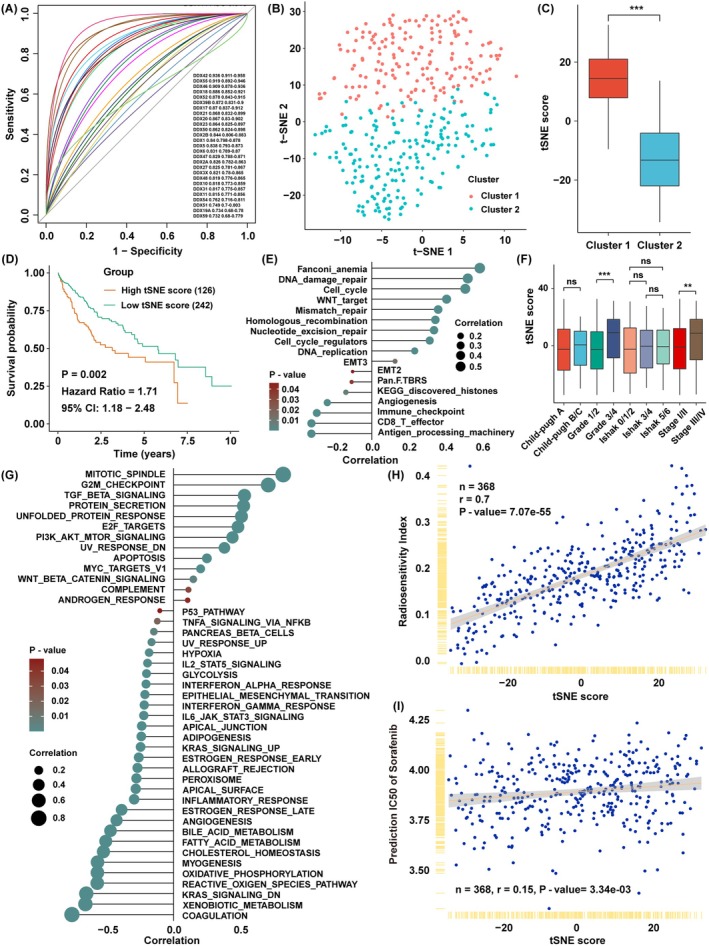
Construction of the *t*SNE score with the expression of DDX family members for HCC. (A) The area under the curve (AUC) of DDX family members between two clusters, the genes with AUC > 0.7 were shown in the right panel. (B) *t*SNE analysis between two clusters with the expression of the genes with AUC > 0.7. (C) The comparison of *t*SNE score between two clusters. (D) Survival analysis of two *t*SNE score groups in HCC. (E) Correlation analysis between the enrichment scores of pathways and the *t*SNE score. (F) The comparison of *t*SNE score between different clinical groups. (G) Correlation analysis between the enrichment scores of pathways and the *t*SNE score. (H) Correlation analysis between the RSI and the *t*SNE score. (I) Correlation analysis between the prediction IC50 of sorafenib and the *t*SNE score. The asterisks represent the statistical *p*‐value (**p < 0.01, ***p < 0.001, ns, no significance).

ROC analysis showed that the AUC of 27 DDX family genes was higher than 0.7 (Figure 2A), with the expression of these genes also able to realise the grouping well via *t*SNE analysis (Figure 2B). Considering the individual heterogeneity and complexity among the HCC samples, a set of scoring systems, *t*SNE score, was generated with the expression of these 27 DDX family genes to quantify the aberration levels. Each cluster displayed different levels of the *t*SNE score, meaning a good reflection of the differences between clusters (Figure 2C). Survival analysis showed that the prognosis of the high *t*SNE score group was significantly worse than that of the low *t*SNE score group (Figure 2D), suggesting that the *t*SNE score was a risk factor for HCC patients. To verify the correlation between pathway variation and *t*SNE score, GSVA with another pathway signature showed that DNA damage repair and cell cycle‐related pathways were highly positively correlated with *t*SNE score, while antigen processing, CD8 T cell effector, and immune checkpoint pathways were negatively correlated with *t*SNE score (Figure 2E). Moreover, we found that the *t*SNE score was higher in grade III/IV and stage III/IV patients than in grade I/II and stage I/II, indicating that the *t*SNE score might be correlated with tumour initiation and progression (Figure 2F). Therefore, we leveraged GSVA to verify the biological function of the *t*SNE score, showing that the *t*SNE score was significantly positively correlated with several cell survival‐related pathways, such as mitotic spindle and several cell cycle‐related pathways, which were the same as the DDX family clusters displayed above (Figure 2G). Given the relationship between DNA damage repair and the *t*SNE score, we speculated that the *t*SNE score might reflect the ability of DNA damage repair after DNA toxic treatment. Unexpectedly, RSI was positively correlated with the *t*SNE score, indicating that the levels of the *t*SNE score might be related to cell response to radiation therapy (Figure 2H). While the *t*SNE score was slightly correlated with the prediction IC50 of sorafenib, it indicated that the aberration of DDX family members might be correlated with targeted therapy resistance (Figure 2I). These findings supported that the aberrant expression levels of DDX family members might be highly associated with tumour progression and treatment resistance.

### 3.2 Identification of DDX42 as a Candidate Gene Correlated With Tumour Progression and Treatment Resistance in HCC

**FIGURE 3:**
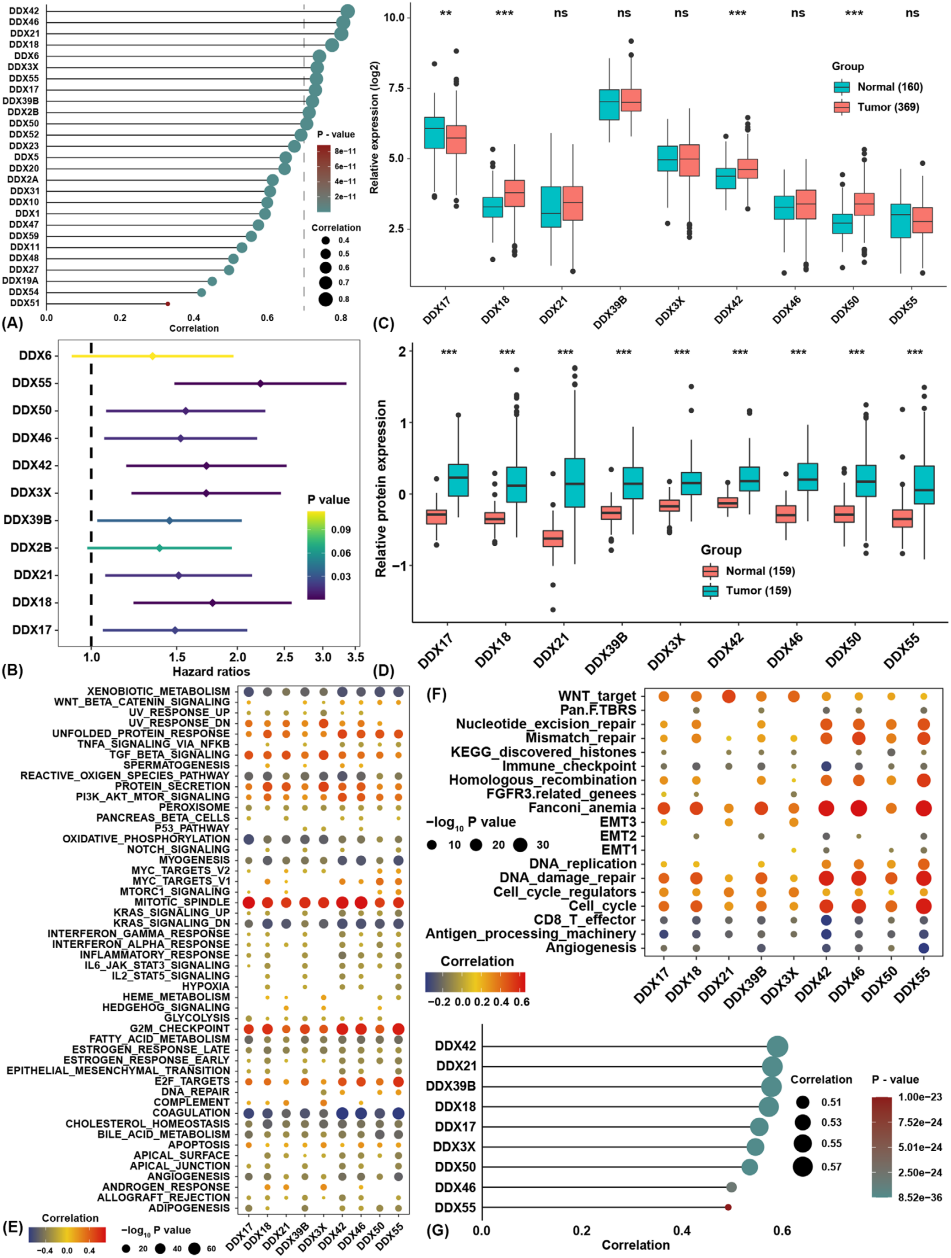
Identification of DDX42 as a candidate gene in HCC. (A) Correlation analysis between the expression of DDX family members involved in the *t*SNE score and the *t*SNE score. (B) Survival analysis of nine DDX family members. (C) The mRNA expression level comparison of nine DDX family members between normal and HCC tissues in TCGA and GTEx datasets. (D) The protein level comparison of nine DDX family members between normal and HCC tissues in PDC. (E) Correlation analysis among the expression of nine DDX family members and the enrichment scores of pathways. (F) Correlation analysis among the expression of nine DDX family members and the enrichment scores of pathways. Dot size represents the −log_10_ *p* value. Blank areas indicate positions where no statistically significant difference in relevance was detected. (G) Correlation analysis between the expression of nine DDX family members and the RSI. The asterisks represent the statistical *p*‐value (**p < 0.01, ***p < 0.001, ns, no significance).

To find homogeneous alternative genes, a correlation analysis was conducted between the expression of DDX family genes involved in the *t*SNE score algorithm and the *t*SNE score (Figure 3A). We found the coefficients of 11 DDX family genes were higher than 0.7, and survival analysis showed that nine of them were risk factors for HCC patients (Figure 3B). Among these nine genes, the transcriptional levels of DDX18, DDX50, and DDX42 were higher in tumours than in normal tissues, while the expression of DDX17 was lower in tumour tissues (Figure 3C). Intriguingly, the protein levels of all these genes were significantly higher in tumours than in normal tissues (Figure 3D). GSVA showed that the expression of these genes was highly positively correlated with the enrichment score of E2F targets, G2M checkpoint, mitotic spindle and PI3K/AKT pathways, while they were highly negatively correlated with coagulation, oxidative phosphorylation and xenobiotic metabolism pathways (Figure 3E). To validate these, GSVA with another pathway signature showed that the expression of these genes was highly positively correlated with cell cycle, DNA damage repair, Fanconi anaemia, and WNT pathways, and merely negatively correlated with CD8 T effector and antigen processing machinery pathways (Figure 3F). These findings indicated that DDX family members might be associated with tumour progression processes, such as cell cycle and mitosis, and treatment resistance, including radiation and target therapies. Correlation analysis showed that DDX42 was the most relevant gene with RSI (Figure 3G), and combined with the above analyses, we eventually chose DDX42 as the candidate gene for further analysis.

**FIGURE 4:**
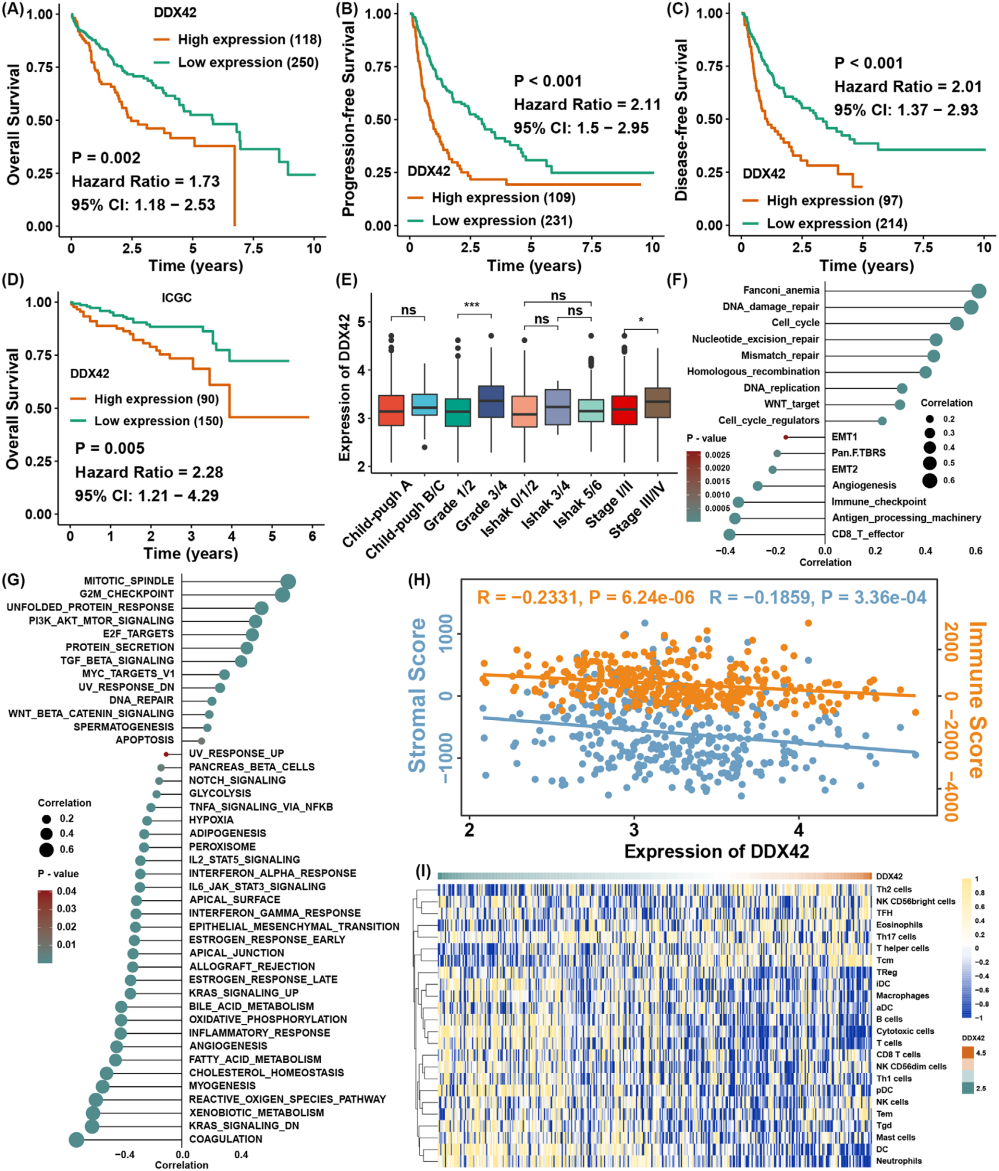
DDX42 might be correlated with tumour progression and treatment resistance in HCC. (A–C) Kaplan–Meier curves of OS, PFS and DFS in HCC patients with different expression levels of DDX42 in TCGA. (D) Kaplan–Meier curves of OS in HCC patients with different expression levels of DDX42 in ICGC. (E) The comparison of DDX42 expression levels between different clinical groups. (F, G) Correlation analysis between the enrichment scores of pathways and the mRNA expression level of DDX42. (H) Correlation analysis between the ESTIMATE score and the mRNA expression level of DDX42. (I) Correlation analysis between the immune cells infiltration levels and the mRNA expression level of DDX42. The asterisks represent the statistical *p*‐value (*p < 0.05, ***p < 0.001, ns, no significance).

Survival analysis showed that higher expression of DDX42 was associated with worse OS, PFS, and DFS in TCGA (Figure 4A–C), and the result in the validation cohort ICGC dataset also showed worse OS in the DDX42 higher expression group (Figure 4D). The transcriptional level of DDX42 was higher in the grade III/IV and stage III/IV group than in the grade I/II and stage I/II group (Figure 4E). GSVA validated that DDX42 was highly positively correlated with cell cycle and DNA damage repair‐related pathways, while DDX42 was negatively correlated with immune‐related pathways (Figure 4F,G). ESTIMATE showed that the expression of DDX42 was negatively correlated with immune and stromal score (Figure 4H), and most immune cell infiltration levels were highly negatively correlated with the expression of DDX42, indicating that DDX42 might play an immune inhibition function in HCC (Figure 4I).

### 3.3 DDX42 Was Correlated With Cell Proliferation, Radiation and Sorafenib Resistance

**FIGURE 5:**
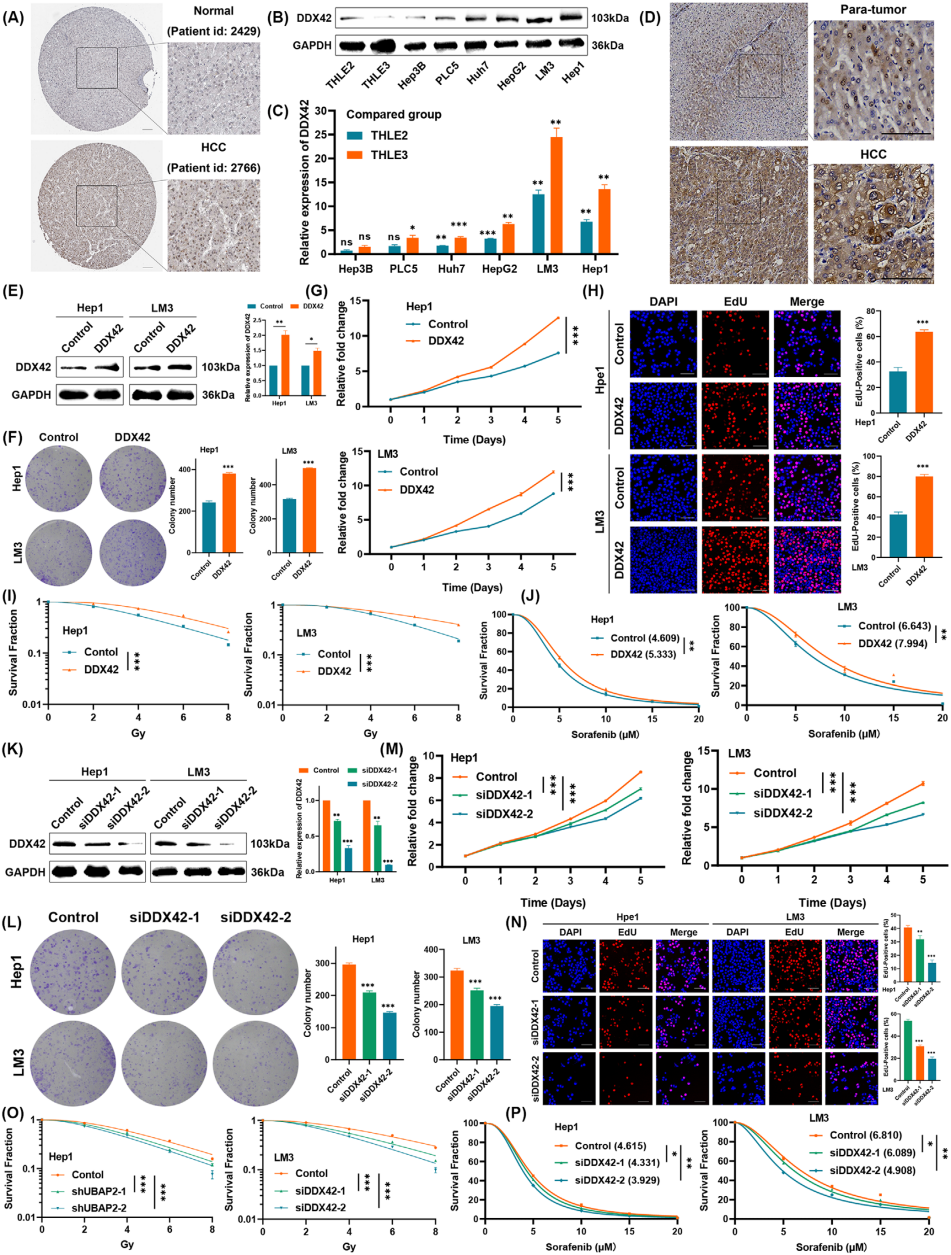
DDX42 was correlated with cell proliferation, radiation and sorafenib resistance. (A) Images of DDX42 in normal and HCC tissues in the HPA dataset (the scale bar represents 100 μm). (B, C) The relative protein and transcriptional levels of DDX42 in different HCC cell lines and normal liver cells. (D) Representative images of immunohistochemistry staining of DDX42 on HCC samples and para‐tumour sections (the scale bar represents 100 μm). (E) WB for DDX42 in HCC cells transfected with DDX42 lentivirus. (F) Overexpression of DDX42 promotes colony formation. (G) CCK‐8 assays for the indicated HCC cells overexpressed of DDX42 over 5 days. (H) EdU assays for the indicated HCC cells overexpressed of DDX42 for 48 h (the scale bar represents 100 μm). (I) Survival fraction with a multi‐target single‐hit model for the indicated HCC cells overexpressed of DDX42. (J) The proliferation for the indicated HCC cells overexpressed of DDX42 after sorafenib treatment was detected by CCK‐8 assay. (K) WB for DDX42 in HCC cells transfected with DDX42 siRNAs. (L) Knockdown of DDX42 inhibits colony formation of HCC cells. (M) CCK‐8 assays for the indicated HCC cells transfected with DDX42 siRNAs over 5 days. (N) EdU assays for the indicated HCC cells transfected with DDX42 siRNAs for 48 h (the scale bar represents 100 μm). (O) Survival fraction with a multi‐target single‐hit model for the indicated HCC cells transfected with DDX42 siRNAs. (P) The proliferation for the indicated HCC cells transfected with DDX42 siRNAs after sorafenib treatment was detected by CCK‐8 assay. Three independent experiments were performed, and the asterisks represent the statistical *p*‐value (*p < 0.05, **p < 0.01, ***p < 0.001 and ns, no significance).

Using the HPA database, we confirmed that the protein level of DDX42 was higher in HCC tissues than in normal liver tissues (Figure 5A). To verify the differential expression of DDX42 in HCC cells, WB and qRT‐PCR were performed on a panel of cell lines, including Hep3B, PLC5, Huh7, HepG2, LM3, Hep1 and normal hepatocytes THLE2 and THLE3 (Figure 5B,C). IHC staining of DDX42 was performed on 10 paired HCC specimens, and the expression of DDX42 was higher in the HCC section than in the para‐tumour section (Figure 5D). To verify the underlying biological function of DDX42 in HCC, we next constructed DDX42 stably expressing cell lines; the efficiency of transfection was validated by WB (Figure 5E). Overexpression of DDX42 could moderately promote cell growth of HCC cells, as shown in colony formation (Figure 5F). Furthermore, the results of CCK‐8 and EdU assays suggested that overexpression of DDX42 markedly enhanced the proliferation abilities of HCC cells (Figure 5G,H). Given the bioinformatics analysis above, we speculated that DDX42 might contribute to radio‐resistance and sorafenib resistance in HCC. Therefore, radiation clonogenic assays showed that DDX42 could increase the ability of HCC cells to radio‐resistance (Figure 5I), and CCK‐8 assay showed that DDX42 significantly enhanced the proliferation of HCC cells exposed to sorafenib treatment (Figure 5J). Moreover, we next silenced DDX42 using siRNAs, and the efficiency of transfection was demonstrated by WB (Figure 5K). As expected, the knockdown of DDX42 moderately inhibited cell growth of HCC cells, as shown in colony formation (Figure 5L). Furthermore, the results of CCK‐8 and EdU assays suggested that knockdown of DDX42 markedly suppressed the proliferation of HCC cells (Figure 5M,N). Radiation clonogenic assays showed that DDX42 knockdown increased radio‐sensitivity in HCC cells (Figure 5O), and CCK‐8 assay showed that DDX42 knockdown significantly enhanced the efficacy of sorafenib (Figure 5P).

### 3.4 DDX42 Contributes to Formation of GRB2 Mature mRNA in HCC

**FIGURE 6:**
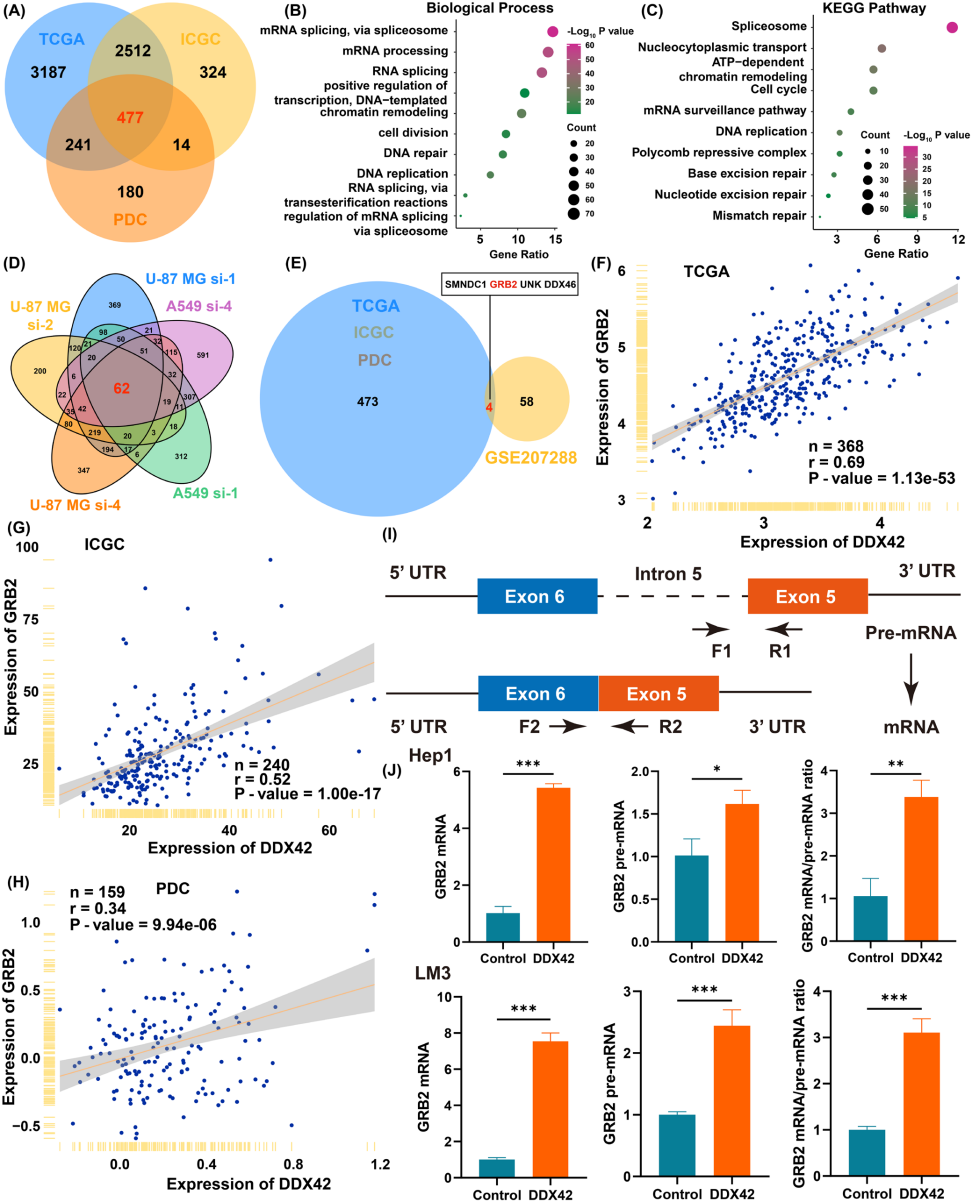
DDX42 contributes to the formation of GRB2 mature mRNA in HCC. (A) Intersection of DDX42 positively correlated genes among TCGA, ICGC and PDC datasets. (B, C) GO and KEGG analysis for intersected genes positively correlated with DDX42. (D) Intersection of genes downregulated by knocking down of DDX42 between U87‐MG and A549 cells. (E) Intersection between DDX42 positively correlated genes and genes downregulated by knocking down of DDX42. (F–H) Correlation analysis between the expression of DDX42 and GRB2 in TCGA, ICGC and PDC datasets. (I) Schematic representation of strategy to identify intron‐exon (unspliced, pre‐mRNA) and exon‐exon (spliced, mature RNA) junction sites using qRT‐PCR. (J) Relative levels of unspliced and spliced GRB2 transcripts and their ratio in LM3 and Hep1 cells stably overexpressed DDX42. The asterisks represent the statistical *p*‐value (*p < 0.05, ** *p* < 0.01, ***p < 0.001).

Given that DDX42 was an important regulator of transcriptional processes, correlation analysis was conducted to find the most relevant genes with DDX42 in transcriptional and protein levels. As a result, 477 positively correlated genes with DDX42 were intersected among TCGA, ICGC, and PDC cohorts (Figure 6A); GO and KEGG analysis showed that genes positively correlated with DDX42 were enriched in mRNA splicing and spliceosome‐related biological functions and pathways (Figure 6B,C). To further investigate the genes regulated by DDX42, we employed GSE207288, a dataset containing the transcriptome data of DDX42‐depleted U87‐MG and A549 cells, to find 62 genes that could be downregulated when knocking down DDX42 (Figure 6D). Intersecting with the preceding 477 correlated genes and 62 downregulated genes, four genes (SMNDC1, GRB2, UNK, DDX46) were selected as candidate genes regulated by DDX42 (Figure 6E). While DDX46 functions as an RNA splicing factor similar to DDX42, detailed reports on the biological functions of SMNDC1 and UNK in cancer are currently limited. Among them, we found that GRB2 was an upstream regulator for the PI3K/AKT pathway, which was consistent with our above bioinformatics analysis for the underlying biological function of DDX42. The mRNA expression of DDX42 was highly positively correlated with the mRNA expression of GRB2 in TCGA and ICGC cohorts (Figure 6F,G); consistently, the protein levels of DDX42 and GRB2 were also positively correlated with each other (Figure 6H). Therefore, we wondered whether DDX42 was required for the maturation of GRB2 pre‐mRNA to mature mRNA. To test this, we measured mature (spliced) and immature (unspliced) GRB2 mRNAs using intron‐exon and exon‐exon junction‐specific primers (Figure 6I). When overexpression of DDX42 elevated mature GRB2 mRNAs in both Hep1 and LM3 cells, GRB2 pre‐mRNAs also upregulated slightly (Figure 6J). Importantly, the relative ratio of GRB2 mRNA/pre‐mRNA was significantly higher with DDX42 overexpression in both the Hep1 and LM3 cells, indicating that the aberrant splicing factor DDX42 led to an accumulation of mature GRB2 RNA.

### 3.5 DDX42 Contributes to GRB2‐Dependent Proliferation and Radiation and Sorafenib Resistance in HCC

**FIGURE 7:**
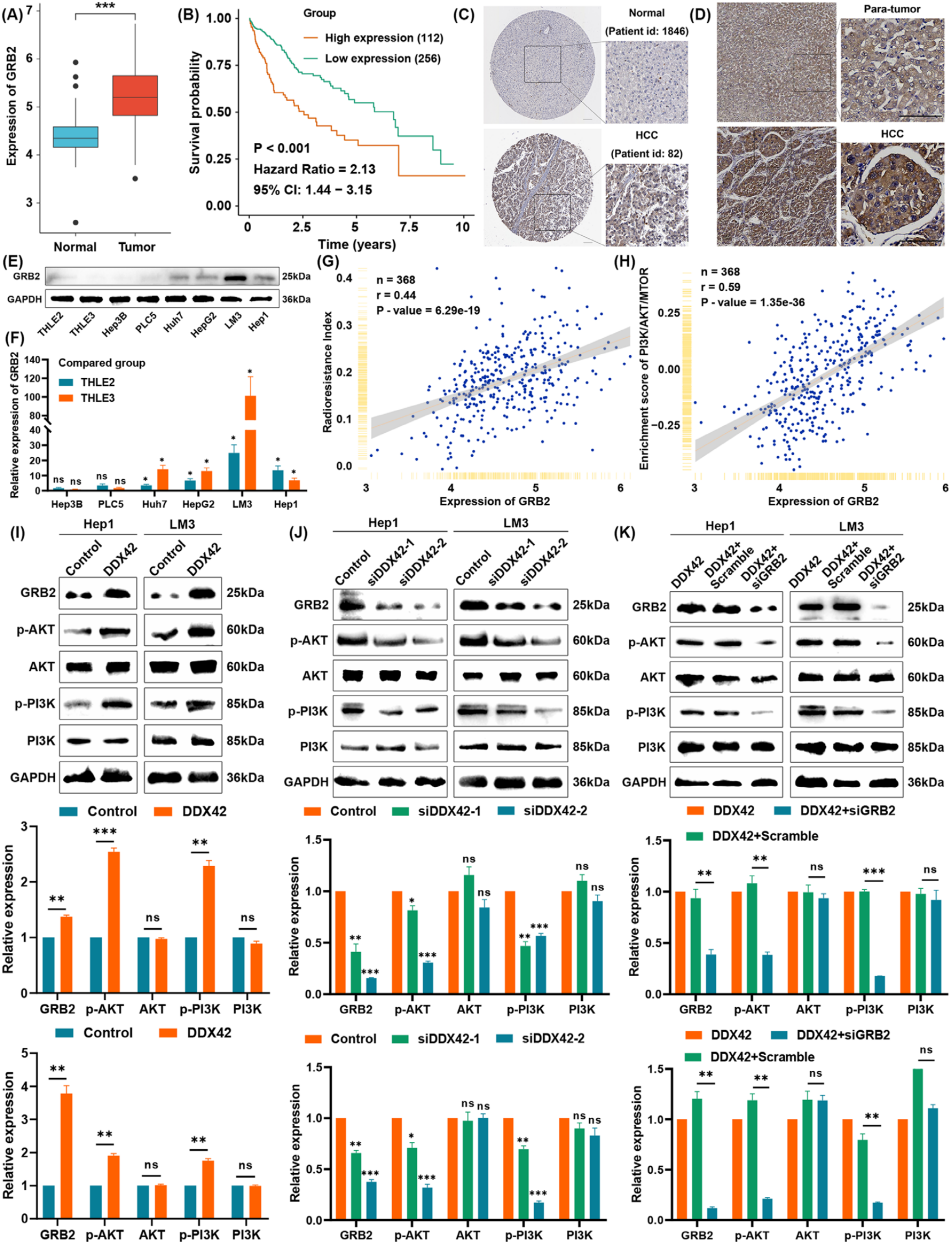
DDX42 activated PI3K/AKT pathways through upregulation of GRB2. (A) The mRNA expression level of GRB2 in HCC and normal tissues in TCGA and GTEx datasets. (B) Kaplan–Meier curves of OS in HCC patients with different expression levels of GRB2 in TCGA. (C) Images of GRB2 in normal and HCC tissues in the HPA dataset (the scale bar represents 100 μm). (D) Representative images of immunohistochemistry staining of GRB2 on HCC samples and para‐tumour sections (the scale bar represents 100 μm). (E, F) The relative protein and transcriptional levels of GRB2 in different HCC cell lines and normal liver cells. (G) Correlation analysis between the expression of GRB2 and RSI in TCGA. (H) Correlation analysis between the expression of GRB2 and the enrichment score of PI3K/AKT pathway in TCGA. (I) Western blot results of GRB2 and PI3K/AKT pathway‐related genes in cells with overexpression of DDX42. (J) Western blot results of GRB2 and PI3K/AKT pathway‐related genes in cells with knockdown of DDX42. (K) Western blot results of GRB2 and PI3K/AKT pathway‐related genes in cells transfected with DDX42 or co‐transfected with GRB2 siRNA. Three independent experiments were performed, and the asterisks represent the statistical *p*‐value (*p < 0.05, **p < 0.01, ***p < 0.001 and ns, no significance).

The mRNA expression level of GRB2 was higher in HCC than in normal tissues (Figure 7A), and the higher expression group of GRB2 displayed worse OS for HCC patients (Figure 7B). Using the HPA database, we confirmed that the protein level of GRB2 was higher in HCC tissues than in normal liver tissues (Figure 7C). IHC staining of GRB2 showed that the expression of GRB2 was higher in the HCC section than in the para‐tumour section (Figure 7D). WB and qRT‐PCR showed that the mRNA and protein levels of GRB2 were higher in HCC cell lines than in normal cell lines (Figure 7E,F). Moreover, we found that the expression of GRB2 was positively correlated with RSI and enrichment score of the PI3K/AKT pathway (Figure 7G,H). Therefore, WB results revealed that overexpression of DDX42 significantly elevated GRB2 and phosphorylated PI3K and AKT (Figure 7I), while suppression of DDX42 resulted in the downregulation of GRB2 and phosphorylated PI3K and AKT (Figure 7J). The elevation of GRB2 and activation of the PI3K/AKT pathway mediated by overexpression of DDX42 could be reversed by the suppression of GRB2 (Figure 7K).

**FIGURE 8:**
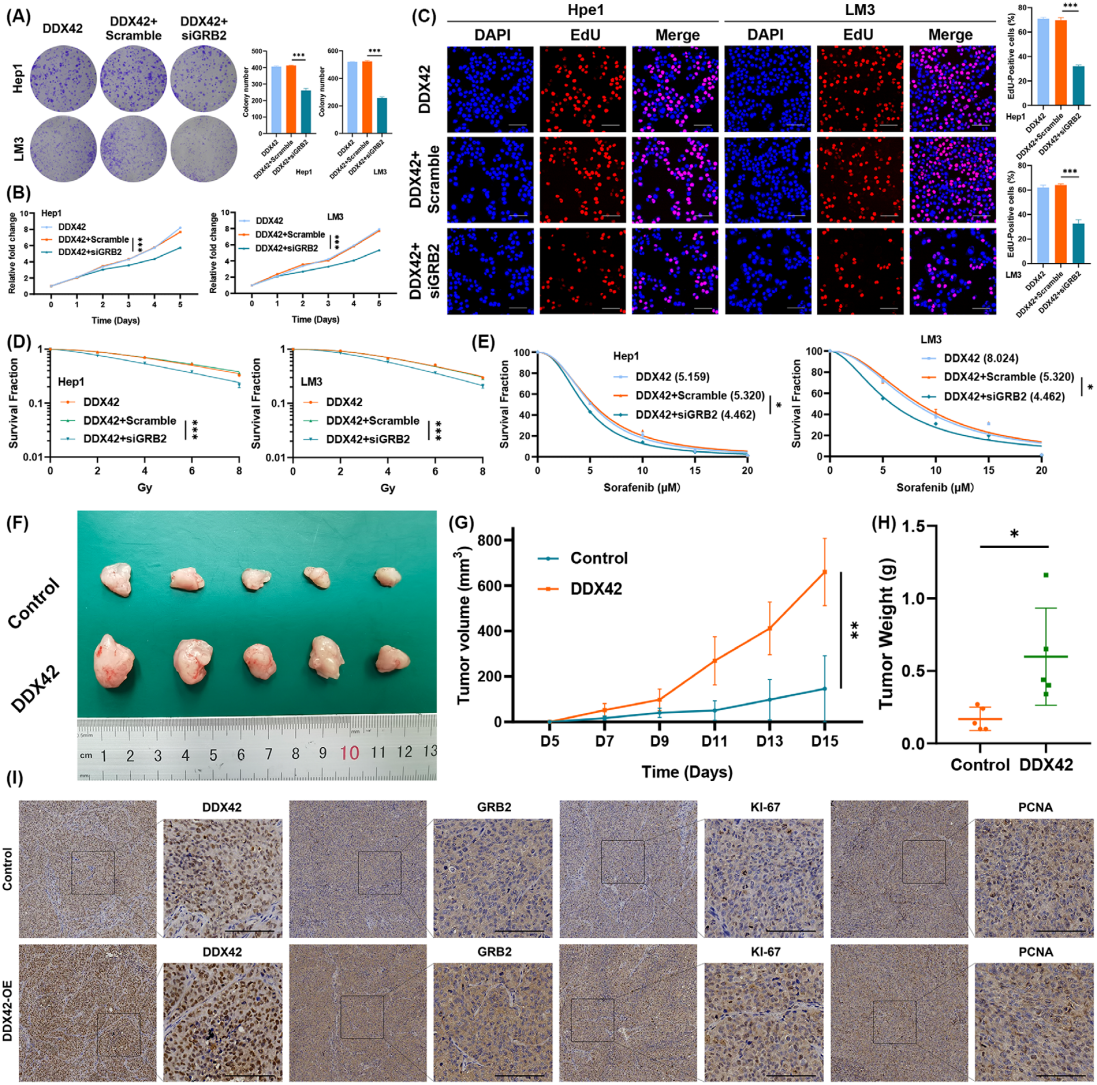
DDX42 contributes to GRB2‐dependent proliferation and radiation and sorafenib resistance in HCC. (A‐C) Colony formation, CCK‐8 and EdU assays in cells transfected with DDX42 or co‐transfected with GRB2 siRNA. The scale bar represents 100 μm. (D) Survival fraction with a multi‐target single‐hit model for the indicated HCC cells transfected with DDX42 or co‐transfected with GRB2 siRNA. (E) The proliferation for the indicated HCC cells transfected with DDX42 or co‐transfected with GRB2 siRNA after sorafenib treatment was detected by CCK‐8 assay. (F) The volumes of subcutaneous tumours in indicated mice. (G, H) Growth curves and tumour weight. Values represent the mean ± SE. (I) Immunohistochemistry staining results of Ki‐67, PCNA, DDX42, and GRB2 in different groups (the scale bar represents 100 μm). The asterisks represent the statistical *p*‐value (*p < 0.05, **p < 0.01, ***p < 0.001).

To further investigate whether the elevation of GRB2 was required for DDX42‐mediated cell proliferation, radiation, and sorafenib resistance in HCC, we downregulated the protein expression of GRB2 in DDX42 overexpression cell lines. We found that inhibition of GRB2 moderately reversed cell growth of HCC cells that expressed ectopic DDX42, as shown in colony formation (Figure 8A). Furthermore, the results of CCK‐8 and EdU assays suggested that suppression of GRB2 markedly suppressed the proliferation of HCC cells (Figure 7B,C). The increased radio‐ and sorafenib resistance by overexpression of DDX42 could be abolished by the suppression of GRB2 using radiation clonogenic and CCK‐8 assays (Figure 7D,E). Altogether, these results indicated that enhancement of GRB2 was required for DDX42‐mediated cell proliferation, radiation, and sorafenib resistance phenotypes in HCC.

A subcutaneous xenograft nude mouse model was utilised to investigate the promotion effects of DDX42 on cell proliferation phenotype in vivo. We found that DDX42 significantly promoted tumour growth as compared to the control group (Figure 8F–H). Further IHC staining revealed that DDX42 could lift the expression of GRB2, KI‐67, and PCNA (cell proliferation markers) in vivo (Figure 8I), as it did in vitro.

## 4 Discussion

**FIGURE 9:**
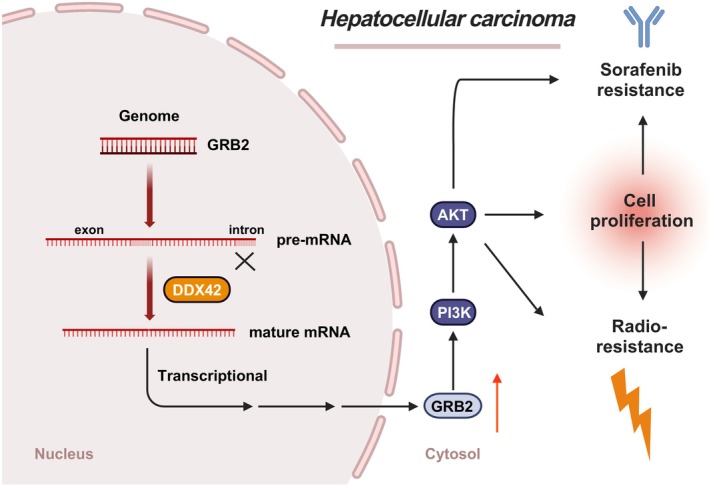
Schematic illustration of the hypothesis for this study.

Our previous studies have shown that gene family members could play different roles in various tumour types [24, 25, 26]. Resorting public datasets with bioinformatics analysis methods could help us to find the most important gene within a gene family in a specific tumour type, which might be powerful to be a diagnostic and therapeutic target. In the present study (Figure 9), we first systematically analysed the DDX family members in HCC for transcriptional and protein levels, and several DDX family members were prognostic factors and differentially expressed in HCC. Using the NMF method, we clustered the HCC samples into two DDX groups with consistent expression patterns, which were highly associated with cell cycle, DNA damage repair, and microenvironment remodelling. To our knowledge, this is the first study that constructed a DDX‐related scoring system based on *t*SNE analysis, which was associated with several biological functions. Using this method, we eventually identified the DDX42 as the candidate DDX family member for further analysis. RSI, a well‐known index to predict radiation sensitivity, and prediction of IC50 for sorafenib were both highly correlated with the expression of DDX42. Based on rigorous experiments in vivo and in vitro, we finalised the basic function of DDX42 on cell proliferation, radiation and sorafenib resistance in HCC.

Although the basic expression and biological function of DDX42 have been investigated in HCC before, several studies have demonstrated that DDX42 could play an important role in regulating the mechanisms of transcriptional and immune response processes [11, 12]. However, based on bioinformatics analysis, we first supposed that DDX42 might be involved in the cell proliferation and treatment resistance phenotypes by urging the maturation process of GRB2, which has been fully understood in several tumour types. In the previous study, several regulating mechanisms have been reported to influence the expression level of GRB2, such as transcriptional and post‐transcriptional levels. NSUN2‐mediated RNA 5‐methylcytosine could promote the stabilisation of GRB2 mRNA [27], and deubiquitinase PSMD14 could stabilise GRB2 at protein level [15]. Here, we reported that the expression level of GRB2 might be regulated by DDX42 by affecting the mRNA maturation level of GRB2.

Previous studies have shown that aberrant expression of splicing factors might affect the RNA maturation process to promote tumorigenesis through the accumulation of mature mRNAs [28, 29]. We found increased expression of GRB2 due to overexpression of DDX42‐mediated mRNA maturation, and GRB2 partially contributes to DDX42 pro‐proliferative function and treatment resistance. Given the multitude of genes impacted by mis‐splicing due to splicing factor dysfunctions, the downstream functional impact is unlikely due to the altered splicing of a single gene variant [30]. Thus, the partial contribution of GRB2 to overall DDX42 function in our study indicates that DDX42 exerts its functions through numerous downstream effector genes. Therefore, systematic transcriptome analysis might be leveraged in further research to analyse the target genes of DDX42 in tumour progression.

After years of clinical practice, radiotherapy and sorafenib treatment indeed could benefit patients with unresectable or advanced HCC as promising local and systemic tumour control strategies, but the effectiveness is weakened by treatment resistance [5, 31, 32]. In recent years, increasing studies have focused on how to improve the efficacy of radiotherapy and targeted therapy in HCC. In this study, we first proved the function of DDX42 and GRB2 in radiation and sorafenib resistance processes, indicating that targeting this axis might be a novel method to conquer the treatment resistance. Moreover, we found that DDX42 might be involved in immune cell infiltration patterns in HCC, and the expression level of DDX42 might be associated with the microenvironment remodelling. Previous studies have shown that DDX46 could inhibit innate immunity by entrapping m6A‐demethylated antiviral transcripts in the nucleus [33], DDX5 could inhibit type I IFN production by promoting degradation of TBK1 [34], and DDX1 might be a prognostic biomarker and correlate with immune infiltrations in HCC [35]. However, the function of DDX42 in regulating immune cells still needs to be further explored.

It is worth emphasising that the prognostic and therapeutic value of DDX42 might present a promising method for HCC treatment. However, practical targeted molecule drugs remain to be developed, and a clinical cohort is also needed to prove the prognostic and diagnostic value of DDX42. We aimed to investigate the combined therapy probabilities of inhibition of DDX42 in vivo for HCC, such as immunotherapy, radiotherapy, and targeted therapy. However, the sample size and absence of clinical features constitute the limitations of our work, which we would improve in further study. However, elucidating the molecular mechanism of tumour progression and treatment resistance is still an urgent need to develop more effective anti‐HCC drugs.

## 5 Conclusions

Collectively, we systematically analysed the DDX family members in HCC and constructed a *t*SNE scoring system to find the most important candidate gene. Based on biological function enrichment analysis, RSI, and prediction IC50 index of sorafenib, we ultimately ascertain the candidate gene DDX42 for further analysis. Importantly, DDX42 overexpression promotes cell proliferation, radiation and sorafenib resistance in HCC cells and activates the PI3K/AKT pathway. Mechanically, DDX42 could urge the mRNA maturation of GRB2, contributing to cell proliferation and enhancement of resistance ability to radiotherapy and sorafenib for HCC cells. In conclusion, our findings facilitate the acknowledgment of tumour initiation and mechanisms of treatment resistance in HCC, and targeting the axis of DDX42 and GRB2 may be promising strategies for synergy with radiotherapy or sorafenib for HCC patients.

## Author Contributions

**Zijian Liu:** conceptualization (lead), data curation (lead), funding acquisition (equal), visualization (lead), writing – original draft (lead). **Jingsheng Yuan:** conceptualization (equal), data curation (equal), funding acquisition (equal), validation (lead). **Fei Liu:** data curation (equal), funding acquisition (equal), validation (equal). **Qiwen Zeng:** data curation (equal), validation (equal). **Zhenru Wu:** data curation (equal), validation (equal). **Jian Yang:** conceptualization (equal), project administration (lead), writing – review and editing (lead).

## Ethics Statement

The Study Using Clinical Samples Was Approved by the Ethics Committee on Biomedical Research, West China Hospital of Sichuan University (2016, No. 120). Informed Consent Was Obtained From all Patients or Their Relatives. All Operations of Experimental Animals Were Performed in Accordance With the National Institutes of Health's Guide for the Care and Use of Laboratory Animals. All Operations Were Approved by the Animal Care and Use Committee of West China Hospital of Sichuan University (2020351A).

## Conflicts of Interest

The authors declare no conflicts of interest.

## Supporting information


**Figure S1:** The various parameter indicators of NMF clustering. Each small graph represents a simulated curve of a specific parameter indicator.

## Data Availability

All data included in this study are available upon request by contact with the corresponding author.
