# Supplementary figures and images for: DDX42 Enhances Hepatocellular Carcinoma Cell Proliferation, Radiation and Sorafenib Resistance via Regulating GRB2 RNA Maturation and Activating PI3K/AKT Pathway
Source: J Cell Mol Med. 2025 Aug 19;29(16):e70793. doi: 10.1111/jcmm.70793 (PMC12364721; doi:10.1111/jcmm.70793)

# NMF rank survey

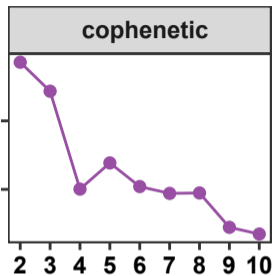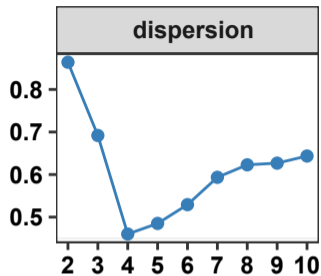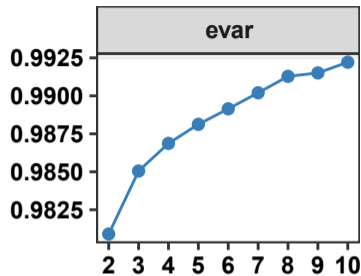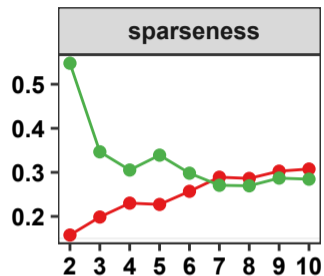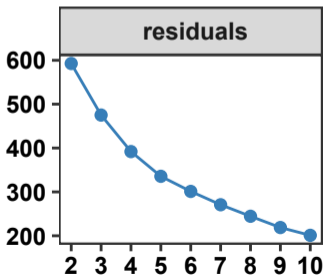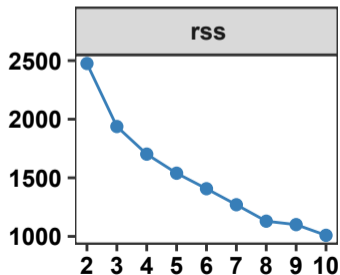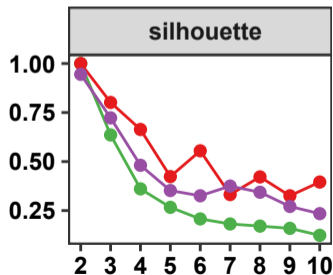

## Measure type

- Basis
- Best fit
- Coefficients
- Consensus
- NA

Factorization rank

Supplement: Supplementary file 1 — Figure S1: The various parameter indicators of NMF clustering. Each small graph represents a simulated curve of a specific parameter indicator. [file JCMM-29-e70793-s001.pdf]
